# Histone lysine methyltransferase G9a is a novel epigenetic target for the treatment of hepatocellular carcinoma

**DOI:** 10.18632/oncotarget.15528

**Published:** 2017-02-20

**Authors:** Masayuki Yokoyama, Tetsuhiro Chiba, Yoh Zen, Motohiko Oshima, Yuko Kusakabe, Yoshiko Noguchi, Kaori Yuki, Shuhei Koide, Shiro Tara, Atsunori Saraya, Kazumasa Aoyama, Naoya Mimura, Satoru Miyagi, Masanori Inoue, Toru Wakamatsu, Tomoko Saito, Sadahisa Ogasawara, Eiichiro Suzuki, Yoshihiko Ooka, Akinobu Tawada, Masayuki Otsuka, Masaru Miyazaki, Osamu Yokosuka, Atsushi Iwama

**Affiliations:** ^1^ Department of Gastroenterology and Nephrology, Graduate School of Medicine, Chiba University, Chiba, Japan; ^2^ Department of Diagnostic Pathology, Kobe University Graduate School of Medicine, Kobe, Japan; ^3^ Department of Cellular and Molecular Medicine, Graduate School of Medicine, Chiba University, Chiba, Japan; ^4^ Department of General Surgery, Graduate School of Medicine, Chiba University, Chiba, Japan

**Keywords:** epigenetics, hepatocellular carcinoma, G9a, BIX-01294, epithelial mesenchymal transition

## Abstract

Histone H3 lysine 9 dimethylation (H3K9me2) is mainly regulated by the histone lysine methyltransferase G9a and is associated with the repression of transcription. However, both the role of G9a and the significance of H3K9me2 in hepatocellular carcinoma (HCC) cells remain unclear. In this study, we conducted loss-of-function assay of *G9a* using short-hairpin RNA and pharmacological interference. Knockdown of G9a reduced H3K9me2 levels and impaired both HCC cell growth and sphere formation. However, transforming growth factor β1-induced epithelial mesenchymal transition (EMT) was not suppressed by G9a knockdown. Combined analyses of chromatin immunoprecipitation followed by sequencing and RNA-sequencing led to successful identification of 96 candidate epigenetic targets of G9a. Pharmacological inhibition of G9a by BIX-01294 resulted in both cell growth inhibition and induction of apoptosis in HCC cells. Intraperitoneal administration of BIX-01294 suppressed the growth of xenograft tumors generated by implantation of HCC cells in non-obese diabetic/severe combined immunodeficient mice. Immunohistochemical analyses revealed high levels of G9a and H3K9me2 in 36 (66.7%) and 35 (64.8%) primary HCC tissues, respectively. G9a expression levels were significantly positively correlated with H3K9me2 levels in tumor tissues. In contrast, in non-tumor tissues, G9a and H3K9me2 were only observed in biliary epithelial cells and periportal hepatocytes. In conclusion, G9a inhibition impairs anchorage-dependent and -independent cell growth, but not EMT in HCC cells. Our data indicate that pharmacological interference of G9a might be a novel epigenetic approach for the treatment of HCC.

## INTRODUCTION

Epigenetics is defined as a mechanism that regulates gene expression without changes in the underlying DNA sequence [1]. Epigenetic regulation of chromatin structure is achieved through various approaches, including histone modifications, DNA methylation, small and non-coding RNAs, and chromatin remodeling [2]. Among these epigenetic changes, methylation of histone H3 at lysine 4 (H3K4) and histone H3 at lysine 36 (H3K36) is associated with transcriptional activation, while methylation of histone H3 at lysine 9 (H3K9) and histone H3 at lysine 27 (H3K27) is involved in transcriptional repression [3]. Histone lysine methyltransferases (HKMTs) typically contain a SET domain and can transfer methyl groups to specific histone tail lysine residues [4]. SUV39H1, ESET, G9a and GLP function as H3K9-specific HKMTs and the methylation of H3K9 has been shown to play an important role in both gene repression and heterochromatin formation [5]. G9a is also a member of the SUV39H group and is responsible for H3K9 mono- and dimethylation (H3K9me2) [6].

Aberrant epigenetic modifications are frequently observed in a wide range of cancers and are considered to play a central role in carcinogenesis and cancer progression [7]. G9a overexpression has been reported in various types of cancers, including HCC [8, 9]. High levels of G9a expression are also considered to be associated with poor prognosis in some cancers [10]. Bai et al. recently reported that G9a overexpression serves as a predictor of unfavorable survival rates after liver resection in patients with HCC [11]. Although these findings indicate that G9a is a potential therapeutic target for HCC, the role of G9a and biological function of H3K9me2 in HCC remains to be elucidated.

In the present study, we performed loss-of-function analyses of G9a using lentivirus-mediated knockdown of *G9a* in culture. Candidate targets of G9a were investigated by chromatin immunoprecipitation followed by sequencing (ChIP-seq) and RNA-sequencing (RNA-seq). In addition, pharmacological disruption of G9a was conducted in culture as well as *in vivo* xenograft models. Finally, G9a expression levels and H3K9me2 levels in primary HCC surgical samples were determined by immunohistochemical analyses.

## RESULTS

### Basal expression of G9a and H3K9me2 level

**Figure 1:**
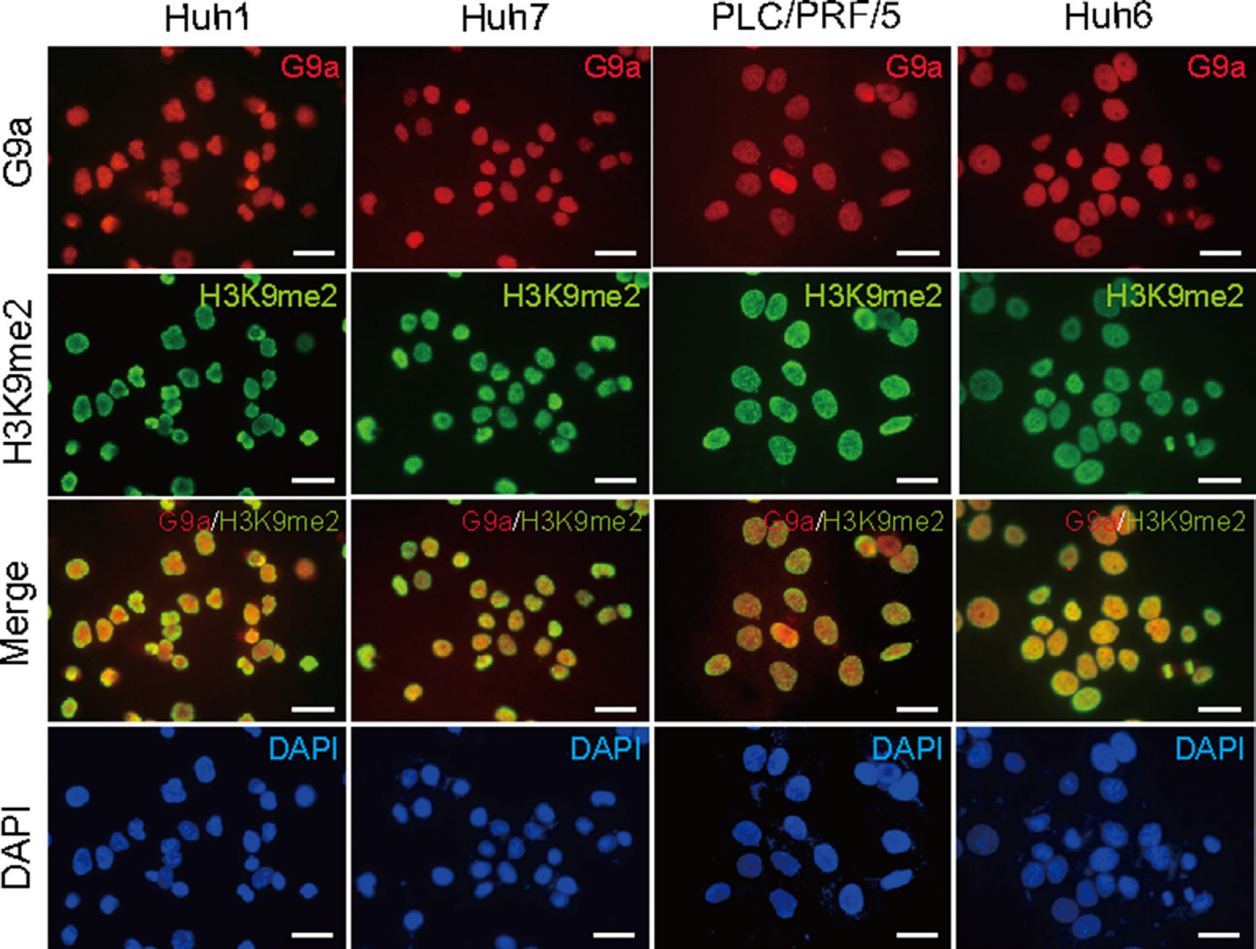
Basal expression levels of G9a and H3K9me2 in Huh1, Huh7, PLC/PRF/5, and Huh6 cells Immunocytochemical analyses for G9a expression (red) and H3K9me2 levels (green) in HCC cells. Nuclear DAPI staining (blue) is also shown. Scale bar = 200 μm.

We first examined the basal expression of G9a and H3K9me2 levels in a number of different HCC cell lines, namely Huh1, Huh7, PLC/PRF/5, and Huh6 cells (Figure 1). Immunocytochemical analyses demonstrated that G9a was highly expressed in the nuclei of almost all HCC cells. Similar to G9a expression, high H3K9me2 levels were also found in the nuclei of HCC cells. These results suggest that G9a and H3K9me2 levels are closely associated in HCC cells.

### Loss-of-function analyses of G9a in HCC cells

**Figure 2:**
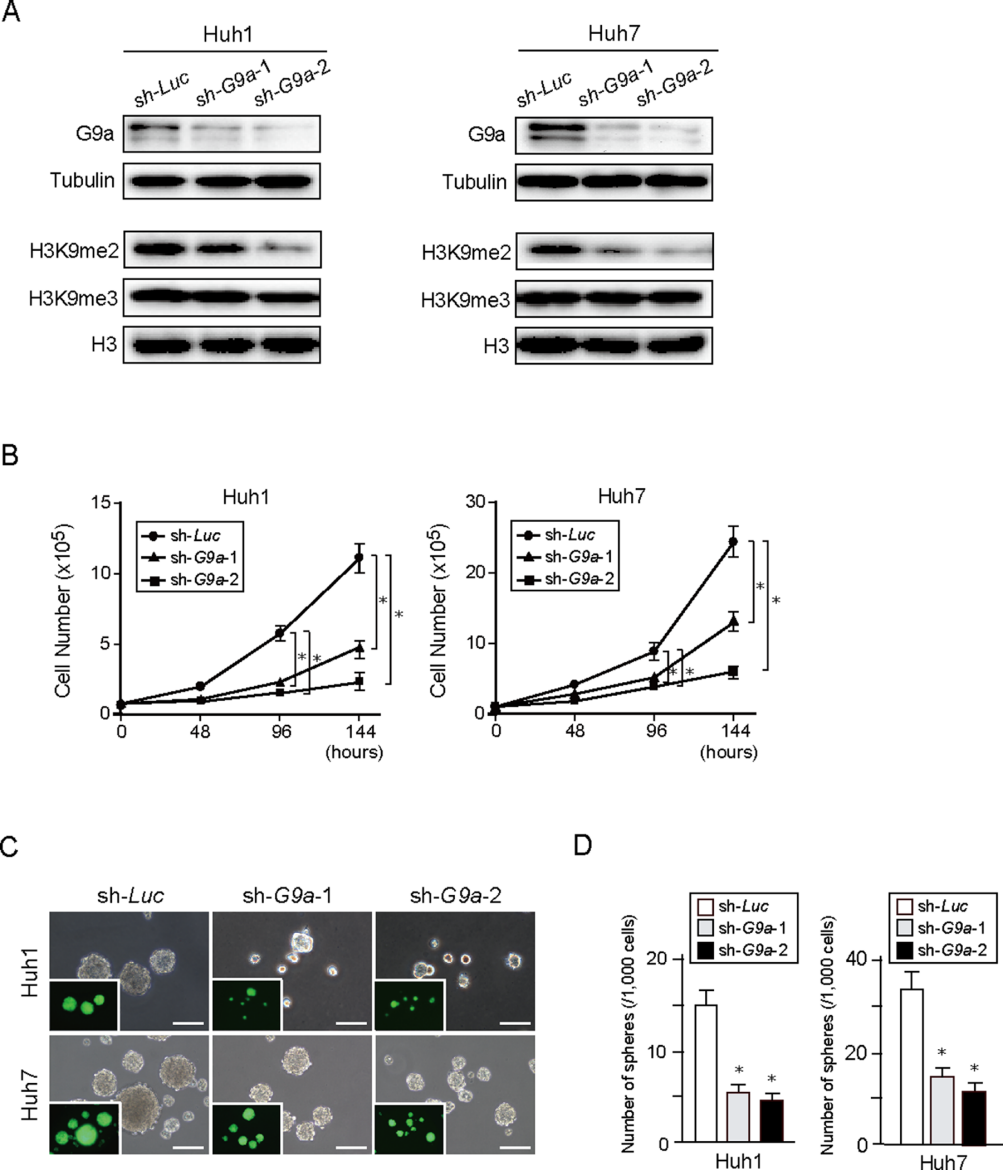
*G9a* knockdown in HCC cells (**A**) Stable knockdown cells were selected by cell sorting for EGFP expression, and subjected to Western blot analysis using anti-G9a, anti-tubulin, anti-H3K9me2, and anti-H3 antibodies. Tubulin and histone H3 were used as loading controls. (**B**) Inhibition of cell proliferation in *G9a* knockdown HCC cells cultured for 48, 96, and 144 hours after seeding. (**C**) Bright-field images of *G9a* knockdown HCC cells showing non-adherent spheres formed after 14 days of culture. Fluorescence images are shown in the insets. Scale bar = 100 μm. (**D**) Number of large spheres generated from 1,000 cells transduced with indicated viruses. Data sets were obtained from three independent experiments. *Statistically significant (*p* < 0.05).

To investigate the role of G9a in HCC cells, we first conducted *G9a* knockdown experiments *in vitro*. We achieved the stable knockdown of *G9a* in Huh1 and Huh7 cells using lentivirus-mediated shRNA directed against *G9a* by enhanced green fluorescent protein (EGFP)-positive cell sorting. A lentiviral vector expressing the shRNA targeted against *luciferase* (*Luc*) was used as a control. Two different shRNAs (sh-*G9a*-1 and sh-*G9a*-2) were able to suppress G9a protein expression as well as reduce H3K9me2 levels (Figure 2A). Although both shRNAs interfered with cell growth and sphere formation, sh-*G9a*-2 showed a more pronounced inhibitory effect than sh-*G9a*-1 (Figure 2B–2D). We then performed xenograft transplantation of sh-*G9a*-2-expressing Huh1 and Huh7 cells using NOD/SCID mice (Supplementary Figure 1). *G9a*-knockdown HCC cell-derived tumors showed delayed onset and slower tumor growth compared with sh-*Luc*-expressing control cell-derived tumors. Altogether, these results indicate that G9a expression and H3K9me2 levels closely affect the cell growth and tumorigenicity of HCC cells.

### Role of G9a in epithelial mesenchymal transition of HCC cells

**Figure 3:**
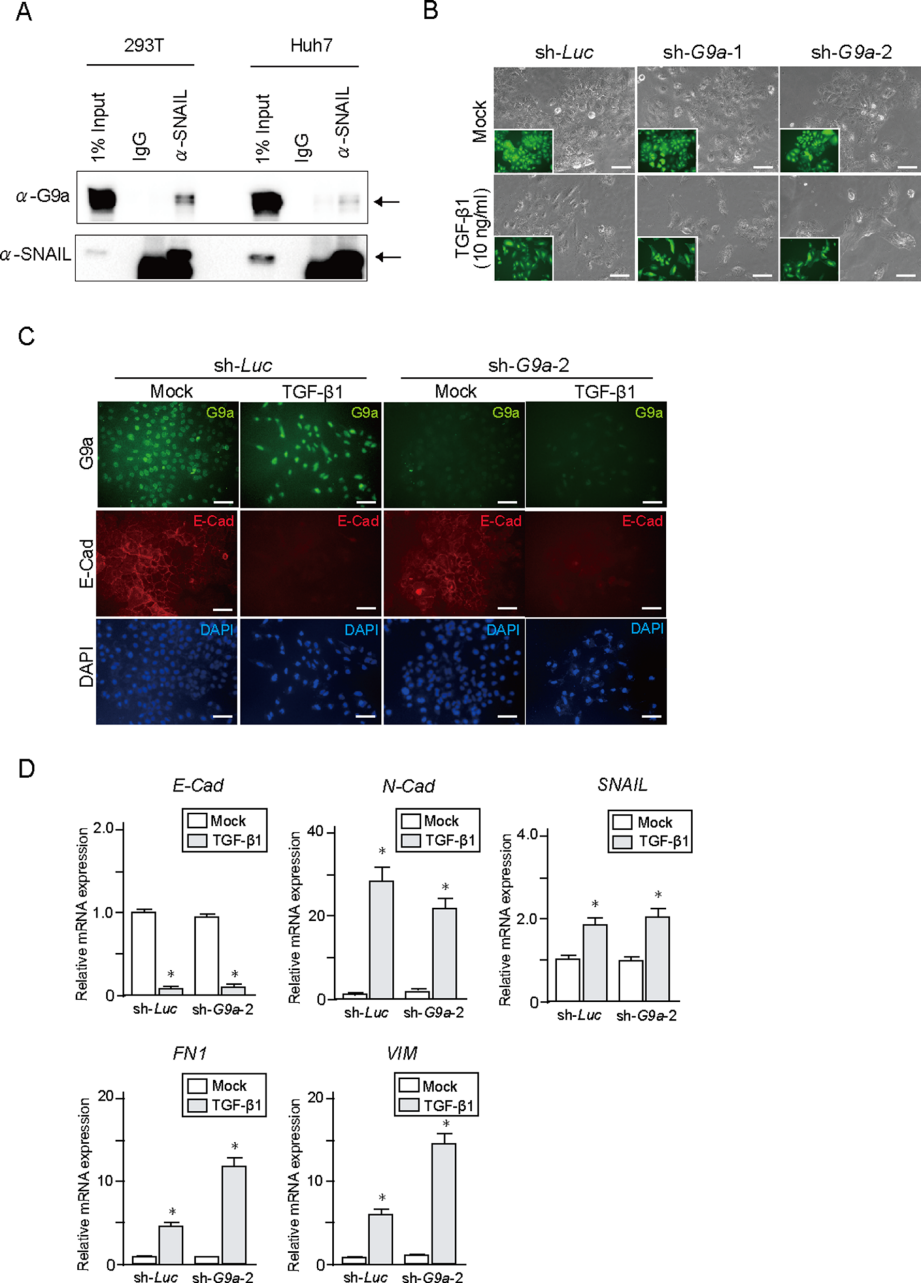
TGF-β1 treatment in *G9a* knockdown HCC cells (**A**) Co-immunoprecipitation of endogenous SNAIL and G9a in Huh7 cells. 293T cells were used as positive control. (**B**) Bright-field images of Huh7 cells treated with TGF-β1 for 48 hours. Fluorescence images are shown in insets. Scale bar = 100 μm. (**C**) Immunocytochemical analyses for G9a (green) and E-cadherin (red) in Huh7 cells. Nuclei stained with DAPI (blue) are also shown. Scale bar = 100 μm. (**D**) Real-time RT-PCR analysis of the expression of EMT markers in Huh7 cells after TGF-β1 treatment for 48 hours. Data sets were obtained from three independent experiments. *Statistically significant (*p* < 0.05).

It has been reported that G9a plays an important role in epithelial mesenchymal transition (EMT) in several cancers such as breast cancer and head and neck squamous cell carcinoma [12, 13]. Interaction between G9a and EMT-related transcription factor SNAIL contributes to E-cadherin repression in human breast cancer cells. Co-immunoprecipitation (co-IP) of endogenous SNAIL and G9a in Huh7 and 293T cells confirmed a physical interaction occurring between G9a and SNAIL (Figure 3A). Therefore, we examined the effect of G9a depletion upon transforming growth factor β1 (TGF-β1)-induced EMT in Huh7 and PLC/PRF/5 cells. Epithelial phenotype of these HCC cells showed no remarkable changes after *G9a* knockdown (Figure 3B and Supplementary Figure 2A and 2B). TGF-β1 treatment successfully changed the epithelial cobblestone morphology to a spindle-like fibroblastic morphology (Figure 3B and Supplementary Figure 2A). However, *G9a* knockdown failed to block TGF-β1-induced EMT in both HCC cells (Figure 3C and Supplementary Figure 2C). Immunocytochemical analyses showed that TGF-β1 treatment reduced expression of E-cadherin in both control and *G9a* knockdown cells (Figure 3C and Supplementary Figure 2B). Concordant with these findings, TGF-β1 treatment of control or G9a knockdown cells resulted in identical degrees of up-regulation of SNAIL as well as mesenchymal markers, including N-cadherin, Fibronectin1, and Vimentin (Figure 3D and Supplementary Figure 2D). In addition, wound healing assay showed no remarkable differences in cellular migration between control and *G9a* knockdown Huh7 cells (Supplementary Figure 3).

Given that miR-200 inhibits TGF-β1-induced E-cadherin downregulation [14], we examined miR-200 role in *G9a* knockdown HCC cells. miR200a and 200b expression levels showed no significant changes after *G9a* knockdown (Supplementary Figure 4A). Furthermore, miR200a overexpression inhibited TGF-β1-induced E-cadherin repression in *G9a* knockdown cells and in control cells. (Supplementary Figure 4B and 4C). Collectively, these results imply that G9a minimally affects TGF-β1-induced EMT in HCC cells and that other regulatory mechanisms that involve miR200 might be more important in EMT induction.

### Exploration of candidate target for G9a

**Figure 4:**
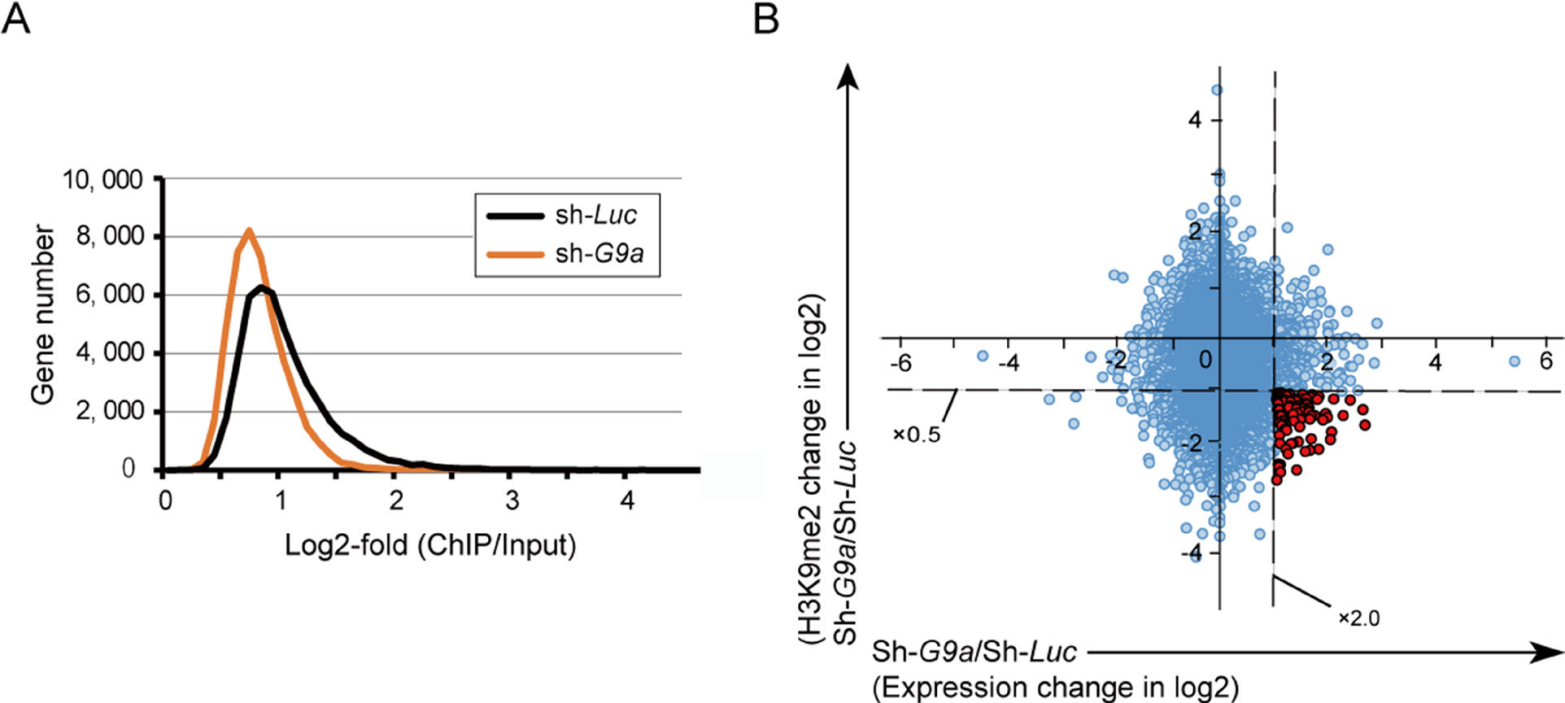
Combined analyses of ChIP-seq and RNA-seq in Huh7 cells (**A**) Number of genes with H3K9me2 enrichment detected by ChIP-seq analysis in *G9a* knockdown cells and control cells. (**B**) Scatter plot depicting the association between H3K9me2 levels and the expression levels of RefSeq genes in *G9a* knockdown cells relative to control cells. The 96 genes showing both decrease in H3K9me2 levels > 2-fold and increase in expression levels > 2-fold are indicated by red dots.

To evaluate the epigenetic changes in *G9a* knockdown, we performed ChIP-seq of H3K9me2 in control and *G9a* knockdown Huh7 cells. ChIP-seq profiling successfully demonstrated that *G9a* knockdown resulted in a marked decrease in H3K9me2 levels in the promoters of coding genes (2.0 kb ± TSS) (Figure 4A). The number of genes with reduced H3K9me2 levels (< 0.5-fold expression) in *G9a* knockdown cells compared with those in control cells, was 4,358.

We next performed RNA-seq for assessing changes in gene expression after *G9a* knockdown. RNA-seq profiling identified 371 upregulated (> 2-fold expression) and 141 downregulated (< 0.5-fold expression) genes. Combined examination of RNA-seq profiling and ChIP-seq data led to successful identification of 96 candidate targets for G9a whose expression was de-repressed (> 2-fold expression) accompanied by a decrease in H3K9me2 levels (< 0.5-fold expression) after *G9a* knockdown (Figure 4B and Supplementary Table 1).

### Pharmacological depletion of G9a in HCC cells

**Figure 5:**
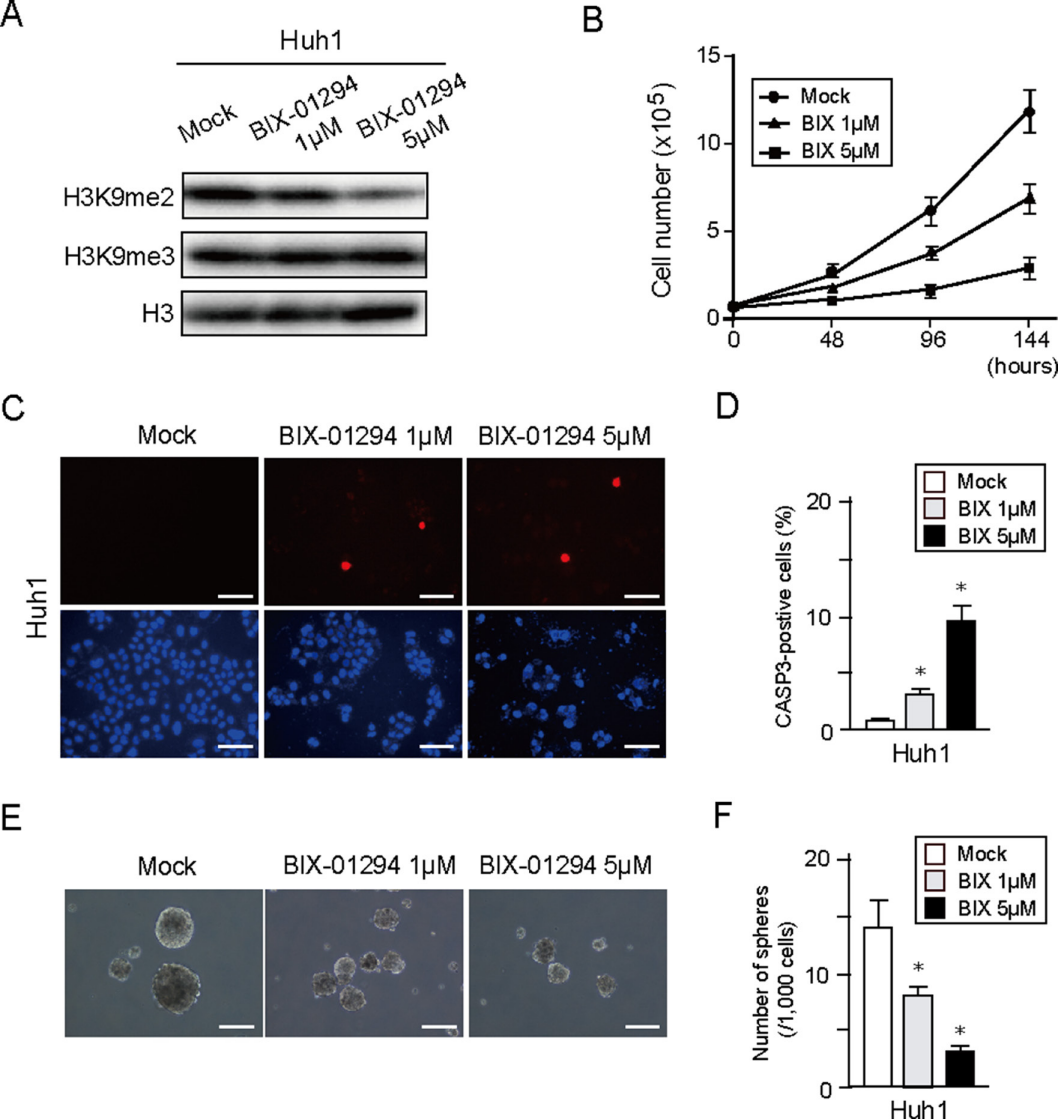
*In vitro* assays of HCC cells treated with BIX-01294 (**A**) Huh1 cells treated or not with BIX-01294 (1 or 5 μM) for 48 hour were subjected to Western blot analysis using anti- H3K9me2 and anti-H3 antibodies. (**B**) Dose-dependent inhibition of proliferation in BIX-01294-treated Huh1 cells. Data sets were obtained from three independent experiments. *Statistically significant (*p* < 0.05). (**C**) Apoptotic cells were identified by immunocytochemical staining for active CASP3 (Red). Nuclear DAPI staining (blue) is also shown. Scale bar = 100 μm. (**D**) Percentage of apoptotic cells. Data sets were obtained from three independent experiments. *Statistically significant (*p* < 0.05). (**E**) Bright-field images showing non-adherent spheres formed by Huh1 cells following 14 days of culture in the presence or absence of BIX-01294. Scale bar = 100 μm. (**F**) Number of large spheres (> 100 μm in diameter) generated from 1,000 cells. Data sets were obtained from three independent experiments. *Statistically significant (*p* < 0.05).

BIX-01294 has been reported to be a small molecule inhibitor of G9a [15]. We have previously demonstrated that BIX-01294 treatment causes decreased levels of H3K9me2 and also inhibition of cell growth and sphere formation in Huh7 cells [16]. We therefore examined whether BIX-01294 showed a similar effect in other HCC cell lines. Western blotting showed that BIX-01294 treatment of Huh1 cells reduced H3K9me2 levels, but not H3K9me3 levels, in a dose-dependent manner (Figure 5A). In addition, BIX-01294 inhibited cell growth and induced apoptotic cell death in a dose-dependent manner (Figure 5B–5D). Similar to these results, non-adherent sphere formation was also suppressed by BIX-01294 treatment (Figure 5E and 5F). Taken together, these results suggest that pharmacological depletion of G9a might exert an anti-cancer effect in HCC cells.

### Effect of BIX-01294 in a xenograft transplantation model

**Figure 6:**
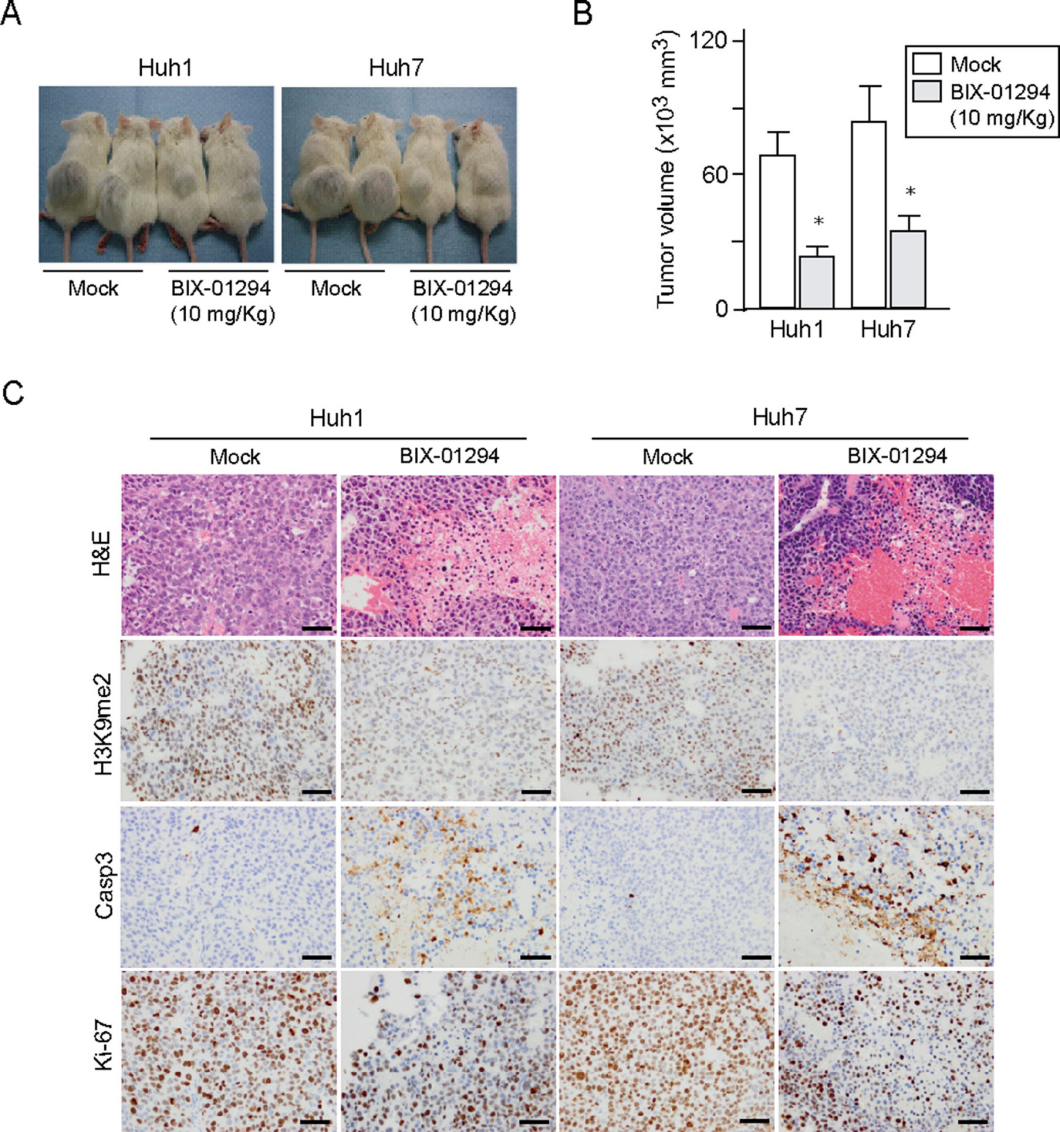
Xenograft transplantation assays in NOD/SCID mice (**A**) A total of 2 × 10^6^ Huh1 or Huh7 cells were transplanted into the subcutaneous space of NOD/SCID mice. Growth of subcutaneous tumors was suppressed by BIX-01294 treatment 8 weeks after transplantation (*N* = 5 per group). (**B**) Volume of subcutaneous tumors was determined at 8 weeks after transplantation (right panel). *Statistically significant (*p* < 0.05). (**C**) Hematoxylin and eosin (H&E) staining and immunohistochemical analyses of representative samples of subcutaneous tumor.

To further explore the anti-cancer potential of G9a inhibition, we examined the anti-cancer effect of the G9a inhibitor BIX-01294 *in vivo* using xenograft transplant models in non-obese diabetic/severe combined immunodeficient (NOD/SCID) mice. Following implantation of 2 × 10^6^ Huh1 or Huh7 cells into NOD/SCID mice, intraperitoneal administration of BIX-01294 (10 mg/Kg) to recipient mice was started immediately. As a result, tumor growth was suppressed by BIX-01294 treatment in both Huh1 and Huh7 implanted mice (Figure 6A and 6B). Immunohistochemical analyses of subcutaneous tumors showed that BIX-01294 significantly reduced H3K9me2 levels (Figure 6C). Immunostaining for Ki-67 and CASP3 also revealed that BIX-01294 treatment inhibited cell growth and induced apoptosis (Figure 6C). We then conducted a non-adherent sphere formation assay with subcutaneous tumor-derived cells. BIX-01294 treatment significantly impaired sphere formation (Supplementary Figure 5). These findings again support the possibility that BIX-01294 could be an effective therapeutic agent for HCC.

### G9a expression and H3K9me2 level in primary HCC

**Figure 7:**
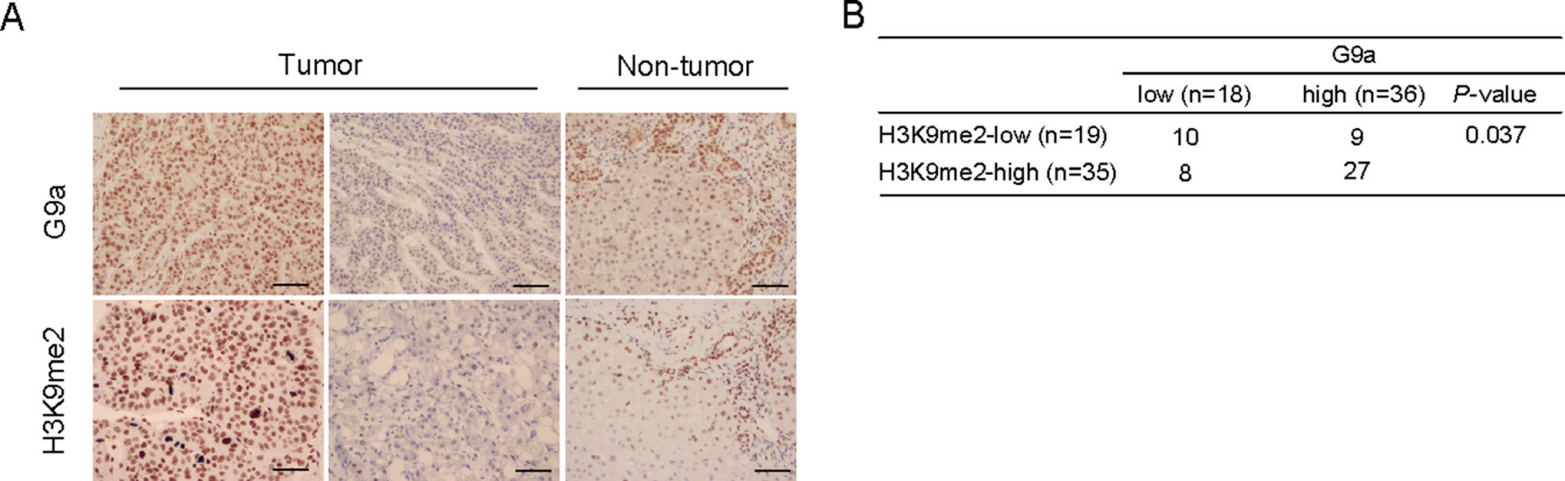
G9a expression and H3K9me2 levels in HCC surgical samples (**A**) Representative immunohistochemical staining for G9a and H3K9me2 in tumor and non-tumor tissues. Scale bar = 100 μm. (**B**) Positive correlation between G9a expression and H3K9me2 levels.

To begin to understand the possible relevance of G9a to primary HCC, we conducted immunohistochemical analyses to examine G9a expression and H3K9me2 levels in 54 primary HCC tissues and corresponding non-tumor tissues. Tumor samples showed varying degrees of G9a expression and varying levels of H3K9me2 (Figure 7A). In contrast, G9a and H3K9me2 were weakly expressed in biliary epithelial cells, infiltrated lymphocytes, and periportal hepatocytes in non-tumor tissues. For G9a, 18 (33.3%) and 36 (66.7%) of 54 HCC samples were classified as G9a^low^ and G9a^high^, respectively. Similarly, for H3K9me2, 19 (35.2%) and 35 (64.8%) of 54 HCC samples were classified as H3K9me2^low^ and H3K9me2^high^, respectively. Importantly, the number of G9a^low^H3K9me2^low^ and G9a^high^H3K9me2^high^ tumors were found to be 10 (18.5%) and 27 (50.0%), respectively (Figure 7B). Fisher's exact test revealed that there was a statistically significant relationship between G9a expression and H3K9me2 levels (*p* <0.05).

## DISCUSSION

G9a is a member of the SET domain containing Suv39 family and catalyzes the dimethylation of histone H3K9 [17]. Subsequent recruitment of heterochromatin protein 1 to H3K9me2 leads to transcriptional silencing [18]. Intriguingly, different histone modifications often show crosstalk in the regulation of gene expression. Ezh2 is a core component of polycomb repressive complex 2 and catalyzes the trimethylation of histone H3K27 [19]. A recent study showed that G9a and Ezh2 share genomic targets and interact to bring about transcriptional repression of developmental genes in mouse embryonic stem cells [20]. In addition, G9a co-localizes with DNA methyltransferase 1 (DNMT1) at H3K9me2 sites and cooperates with DNMT1 in transcriptional repression [21]. Therefore, H3K9me2 established by G9a plays a crucial role in epigenetic transcriptional gene silencing.

To gain insights into the role of G9a in HCC cells, we performed loss-of-function assays in cultured cells. *G9a* knockdown decreased H3K9me2 levels, but not H3K9me3 levels. In line with previous reports, *G9a* knockdown interfered with cell proliferation and tumorigenicity in HCC cells. Recently, inhibition of G9a has been shown to induce not only apoptosis, but also autophagic cell death via the suppression of mTOR pathway [22, 23]. Autophagy is a cellular pathway for the clearance of damaged organelles, and autophagic cell death is one of the important mechanisms of cell death [24]. Further analyses are necessary to elucidate the role of G9a in HCC cell survival.

EMT is a process in which epithelial cells lose their characteristics while acquiring the properties of mesenchymal cells. There is increasing evidence that EMT is deeply involved in the initiation of metastasis for cancer progression [25]. Recent studies have revealed that G9a is closely associated with cell migration and invasion by regulating EMT in several cancers [12, 13]. *In vitro* assays showed that *G9a* depletion failed to cancel TGF-β1-induced EMT in Huh7 and PLC/PRF/5 cells, which have an epithelial cell morphology. However, co-IP assays on Huh7 cells showed an interaction between G9a and SNAIL. These findings suggest that the role of G9a in regulating EMT differs with the cancer types. Further analyses would be necessary to explore the subject.

In this study, combined analyses of ChIP-seq and RNA-seq successfully identified 96 candidate epigenetic targets of G9a. Among these, *p21WAF1/CIP1*, a cyclin-dependent kinase inhibitor, plays an important role in cell cycle arrest [26]. We previously showed that HCC cells treated with histone deacetylase inhibitors increases transcriptional activity and levels of acetylated histone H3 [27]. Taking together, the transcription of *p21WAF1/CIP1* is silenced by histone modifications such as histone methylation and deacetylation in HCC cells. *Tissue inhibitor of metalloproteinase 3* (*TIMP3*) inhibits tumor development via cell cycle arrest and senescence in carcinogen-induced HCC mouse model [28]. Restored expression of these genes followed by *G9a* knockdown might contribute to cell growth inhibition *in vitro* and *in vivo* experiments. In addition, differentiation marker genes such as *cytokeratin 19* (*CK19*) and several *CYP* enzymes were de-repressed after *G9a* knockdown. Given that anchorage-independent growth ability (sphere formation capability) reflects the stemness feature of tumor cells [29], it is possible that the upregulation of these genes was attributable to the decreased number of non-adherent spheres derived from *G9a* knockdown cells.

Recently, a number of HKMT inhibitors have been developed as potential therapeutic cancer agents [30]. Among them is BIX-01294, which is the first specific small molecule inhibitor of G9a. BIX-01294 has been shown to reduce H3K9me2 levels [15]. The mechanism of BIX-01294 inhibition is through competition with G9a substrate, which distinguishes it from other HKMT inhibitors which exert effects through competition with the cofactor S-adenosylmethionine (SAM), the source of the transferred methyl group [31]. It has been reported that BIX-01294 impaired cell growth in other types of cancer [32, 33]. As expected, in the studies reported here, HCC cells treated with BIX-01294 showed reduced H3K9me2 levels. *In vitro* assays revealed that both cell proliferation and sphere formation of HCC cells were significantly impaired by BIX-01294 treatment in a dose-dependent manner. Subsequent xenograft transplantation experiments showed that BIX-01294 suppressed the onset and growth of subcutaneous tumors. Taken together, BIX-01294 appears to exhibit therapeutic value for the treatment of HCC.

Hypoxia is a common characteristic of many solid tumors including HCC [34]. Intratumoral hypoxia usually induces the expression of hypoxia-inducible factor (HIF), which functions as a key regulator which enhances the transcription of genes involved in crucial biological process, including angiogenesis, cell survival, chemoresistance, and invasion [35]. Sorafenib is an oral multikinase inhibitor with anti-angiogenic activity approved for the treatment of advanced HCC [36, 37]. However, the increase in HIF-1 expression in HCC tissues, due to hypoxia, has been shown to contribute to sorafenib resistance [38]. On the other hand, it has recently been reported that hypoxic stress inducers could increase G9a activity and global H3K9me2 levels in several cancer cell lines [39]. Considering these findings, it is possible that the combined use of sorafenib and a G9a inhibitor, such as BIX-01294, could exhibit stronger anti-HCC effects than sorafenib treatment alone.

In conclusion, we have successfully demonstrated that both *G9a* knockdown and pharmacological ablation of G9a reduced H3K9me2 levels and inhibited cell proliferation and tumorigenicity. However, it appeared that G9a exhibited a minimal effect on EMT in HCC cells. Considering that a significant relationship was observed between G9a expression and H3K9me2 levels in primary HCC samples, G9a might be an important epigenetic target in HCC treatment. Further analyses are necessary to elucidate the epigenetic regulatory mechanisms of G9a in HCC cells.

## MATERIALS AND METHODS

### Cell culture and reagents

Human HCC cell lines were obtained from the Health Science Research Resources Bank (HSRRB, Osaka, Japan). For the sphere formation assay, 1,000 cells were plated onto ultra-low attachment 6-well plates (Corning, Corning, NY). The number of spheres (> 100 μm in diameter) was counted following 14 days of culture. For the pharmacological inhibition of G9a, BIX-01294 (Cayman Chemical, Ann Arbor, MI) was used at final concentrations of 1 μM or 5 μM. For the induction of EMT, human TGF-β1 (PeproTech, Rocky Hill, NJ) was used at a final concentration of 10 μg/mL.

### Viral production and transduction

Lentiviral vectors (CS-H1-shRNA-EF-1a-EGFP) expressing short hairpin RNA (shRNA) that targets the human *G9a* (target sequence: sh-*G9a*-1, 5′- GGAT GCTTCTGAAGCTCAAGA -3′; sh-*G9a*-2, 5′- GGTCT TCATGCTGCACCAAGA -3′) and *luciferase* *(Luc)* were constructed. Recombinant lentiviruses were produced as described elsewhere [40]. The cells were transduced with viruses in the presence of protamine sulfate (10 μg/mL). Huh7 cells that stably express miR200a were established by transduction with lentiviral vectors (pLV-miRNA) expressing miR200a (Biosettia Inc., San Diego, CA) and selection with puromycin.

### Cell growth and migration

HCC cell proliferation was assessed using trypan blue staining after 48, 96 and 144 hours in culture. Cell migration was assessed by the scratch-wound assay.

### Western blotting and immunoprecipitation

*G9a*-knockdown cells were selected by cell sorting for EGFP expression. These cells and BIX-01294-treated HCC cells were subjected to Western blot analysis using anti-G9a (Cell Signaling Technology, Danvers, MA), anti-tubulin (Oncogene Science, Cambridge, MA), anti-histone H3K9me2 and anti-histone H3 (Millipore, Billerica, MA) antibodies. Endogenous SNAIL was immunoprecipitated and subjected to Western blotting with rabbit anti-SNAIL (Cell Signaling Technology) and mouse anti-G9a (Abcam, Cambridge, MA) antibodies.

### Immunocytochemistry

Cells were fixed with 2% paraformaldehyde and blocked with normal goat serum. Cells were then stained with anti-G9a (Cell Signaling Technology), anti-E-cadherin (Cell Signaling Technology), anti-caspase 3 (CASP3; Chemicon, Temecula, CA), and anti-H3K9me3 (Millipore) antibodies, followed by incubation with Alexa 555- or Alexa 488-conjugated immunoglobulin G (IgG) (Molecular Probes, Eugene, OR) antibodies. Cells were coverslipped using a mounting medium that contained 4′,6-diamidino-2-phenylindole dihydrochloride (DAPI; Vector Laboratories, Burlingame, CA).

### Quantitative real-time PCR

Quantitative real-time PCR was performed with an ABI PRISM 7300 Sequence Detection System (Applied Biosystems, Foster City, CA) using the Universal Probe Library System (Roche Diagnostics, Mannheim, Germany) according to the manufacturer's directions. The sequences of primers are listed in Supplementary Table 2. For the evaluation of miRNA expression, miRNA-specific TaqMan miRNA assays (Applied Biosystems) for miR-200a, miR-200b, and U6 (internal control) were performed according to the manufacturer's directions.

### RNA-sequencing

RNA-Seq was performed as previously described [41]. In brief, total RNA was subjected to reverse transcription and amplified through 16 cycles with SMARTer Ultra Low Input RNA Kit for Sequencing v3 (Clontech). After sonication, libraries were generated using 50 ng fragmented DNA with a NEBNext Ultra DNA Library Prep Kit (New England BioLabs). After quantification of the libraries with a high-sensitivity Chip using Bioanalyzer (Agilent), sequencing of the samples was performed using a Hiseq1500 (Illumina). Gene expression values were calculated as reads per kilobase of exon unit per million mapped reads using Cufflinks (version 2.2.1).

### ChIP-sequencing

ChIP sequencing (ChIP-Seq) was also performed as previously described [42]. In brief, 1 × 10^7^ Huh7 cells were subjected to immunoprecipited using anti-H3K9me2 (Millipore, Billerica, MA) antibodies. To evaluate the histone modification H3K9me2 in each gene, normalized tag numbers in the region from 2 kb upstream to 2 kb downstream of the transcription start site (TSS) were separated by reads per million (RPM) mapped reads of the corresponding input. The RPM values of the sequenced read were calculated using BEDTools to visualize with Integrative Genomics Viewer (http://www.broadinstitute.org/igv).

### Xenograft transplantation

NOD/SCID mice (Sankyo Laboratory Co. Ltd., Tsukuba, Japan) were bred and maintained in accordance with our institutional guidelines for the use of laboratory animals. A total of 2 × 10^6^ HCC cells stably expressing shRNA against *G9a* or luciferase were implanted into the subcutaneous space on the right and left sides of the backs of recipient NOD/SCID mice, respectively. In the BIX-01294 treatment model, a total of 2 × 10^6^ Huh1 or Huh7 cells were implanted into the subcutaneous space on the backs of NOD/SCID mice. BIX-01294 (10 mg/Kg) was administered intraperitoneally 3 times a week. Tumor formation and growth were observed weekly. Subcutaneous tumors were also subjected to hematoxylin and eosin (H&E) staining and immunohistochemistry with anti-H3K9me2, anti-CASP3, and anti-Ki67 (DAKO, Carpinteria, CA) antibodies. For the analyses of xenograft tumors, small pieces of tumor were digested in DMEM containing 5 mg/mL collagenase type II (Roche). The cell suspension was centrifuged on Ficoll (IBL, Gunma, Japan) to remove dead cells and debris. The harvested cells were subjected to sphere formation assays. All experiments were performed in accordance with the institutional guidelines for the use of laboratory animals.

### Patients and surgical specimens

A total of 54 tumor and non-tumor liver tissue pairs were subjected to histological examination. All patients provided informed consent. They included 41 men and 13 women whose average age was 67.6 years (range: 27–84 years). Paraffin embedded sections of tumors and surrounding non-tumor tissues were examined by H&E staining and immunohistochemistry with anti-G9a and anti-H3K9me2 antibodies. Based on the percentage of cells that strongly expressed these markers, HCC tissues were divided into two groups: G9a^low^ or H3K9me2^low^ (< 50% of tumor cells) and G9a^high^ or H3K9me2^high^ (≥ 50% of tumor cells).

### Statistical analysis

Data are presented as the mean ± standard error of the mean (SEM). Statistical differences between 2 groups were analyzed by either Mann-Whitney *U* test or Chi-square test. Fisher's exact test was used to assess the relationship between H3K9me2 levels and G9a expression. *P* values less than 0.05 were considered significant.

### Accession numbers

Data of RNA-seq and ChIP-seq were deposited in DNA Data Bank of Japan (DRA005376).

## SUPPLEMENTARY MATERIALS FIGURES AND TABLES



## Abbreviations

ChIP-seq: chromatin immunoprecipitation followed by sequencing
DAPI: 4′,6-diamidino-2-phenylindole dihydrochloride
DNMT1: DNA methyltransferase 1
EGFP: enhanced green fluorescent protein
EMT: epithelial mesenchymal transition
H&E: hematoxylin and eosin
HCC: hepatocellular carcinoma
HIF: hypoxia-inducible factor
HKMT: histone lysine methyltransferase
H3K9: methylation of H3 at lysine 9
H3K27: H3 at lysine 27
H3K9me2: H3K9 dimethylation
IP: immunoprecipitation
NOD/SCID: Non-obese diabetic/severe combined immunodeficiency
RNA-seq: RNA-sequencing
TGF-β1: transforming growth factor β1
