# Supplementary material for: Histone lysine methyltransferase G9a is a novel epigenetic target for the treatment of hepatocellular carcinoma
Source: Oncotarget. 2017 Feb 20;8(13):21315–26. doi: 10.18632/oncotarget.15528 (PMC5400586; doi:10.18632/oncotarget.15528)
Supplement: Supplementary file 1 [file oncotarget-08-21315-s001.pdf]

Histone lysine methyltransferase G9a is a novel epigenetic target for the treatment of hepatocellular carcinoma

Supplementary Materials

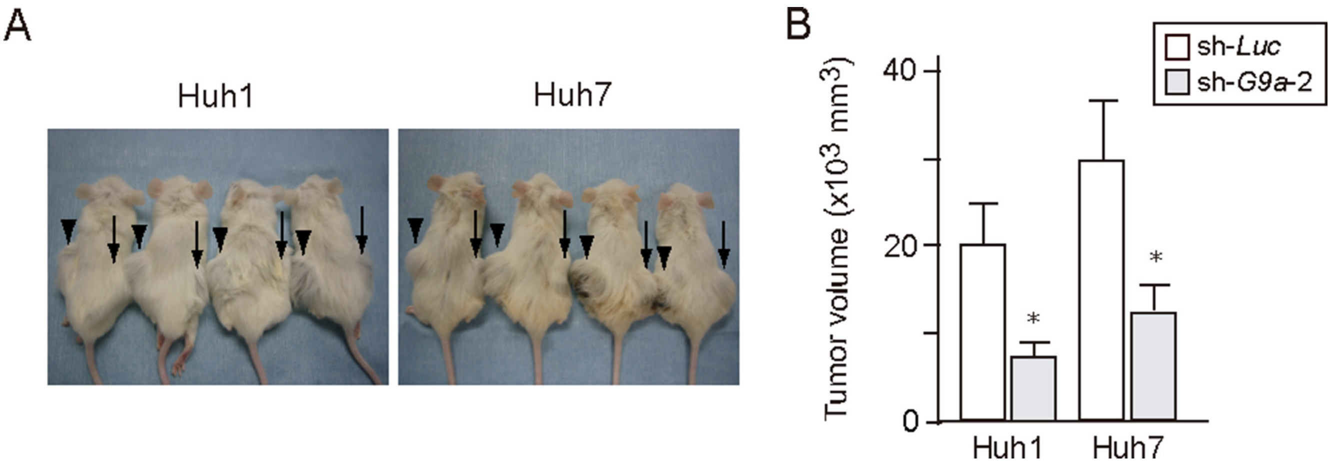

Supplementary Figure 1: Xenograft transplantation in NOD/SCID mice. (**A**) In total, 2×10^6 *G9a* knockdown Huh1 and Huh7 cells were separately implanted into the subcutaneous space of NOD/SCID mice. *G9a* knockdown cells developed apparently smaller tumors in the right subcutaneous space (arrows) than sh-*Luc*-expressing control cells in the left space (arrowheads) 6 weeks after the transplantation ($N = 5$ per group). (**B**) Tumor volume was estimated 6 weeks after transplantation. *Statistically significant ($p < 0.05$).

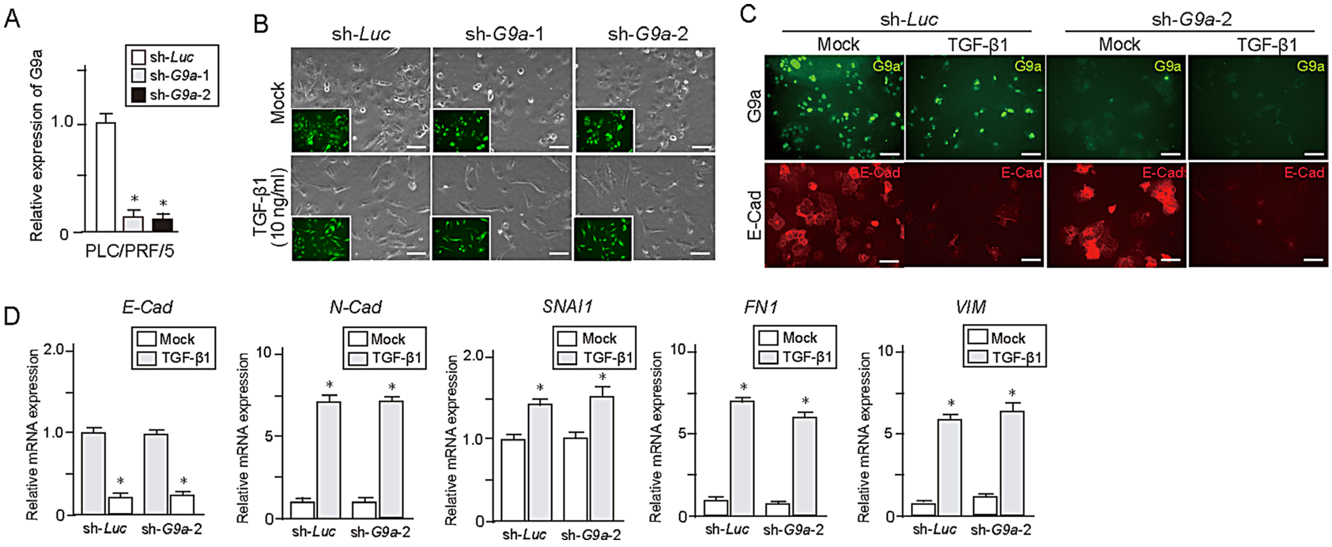

Supplementary Figure 2: TGF-β1-induced EMT in *G9a* knockdown PLC/PRF/5 cells. (**A**) Real-time RT-PCR analysis of G9a expression in stable *G9a* knockdown cells. Data sets were obtained from three independent experiments. *Statistically significant ($p < 0.05$). (**B**) Bright-field images of PLC/PRF/5 cells treated with TGF-β1 for 48 hours. Fluorescence images are shown in insets. Scale bar = 100 µm. (**C**) Immunocytochemical analyses for G9a (green) and E-cadherin (red) expression in PLC/PRF/5 cells. Scale bar = 100 µm. (**D**) Real-time RT-PCR analysis of the expression of EMT markers in PLC/PRF/5 cells after TGF-β1 treatment for 48 hours. Data sets were obtained from three independent experiments. *Statistically significant ($p < 0.05$).

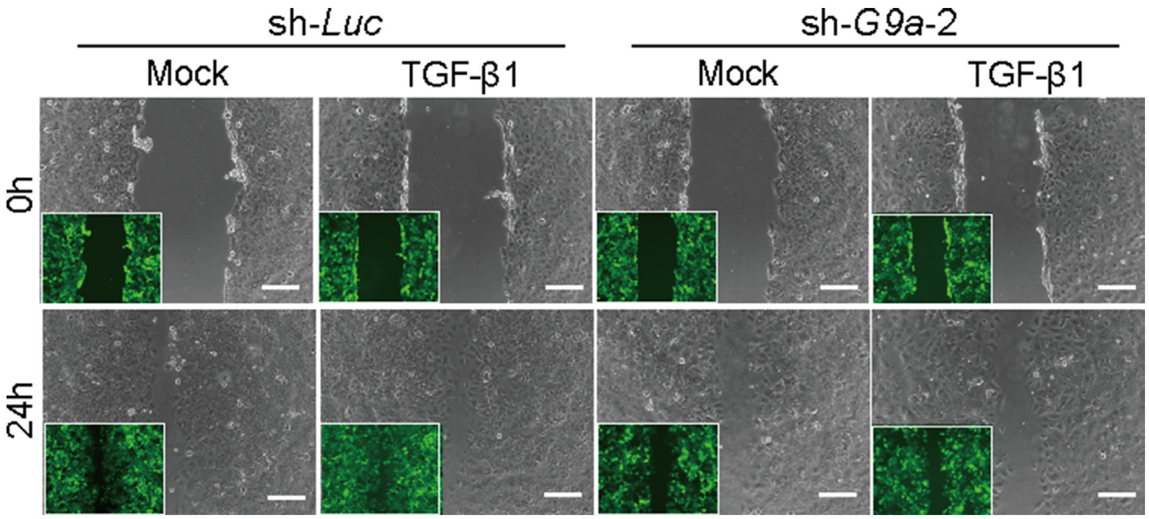

Supplementary Figure 3: Wound healing assay of Huh7 cells. No remarkable differences in migration ability were observed between control and *G9a* knockdown cells after *TGF*-β1 treatment.

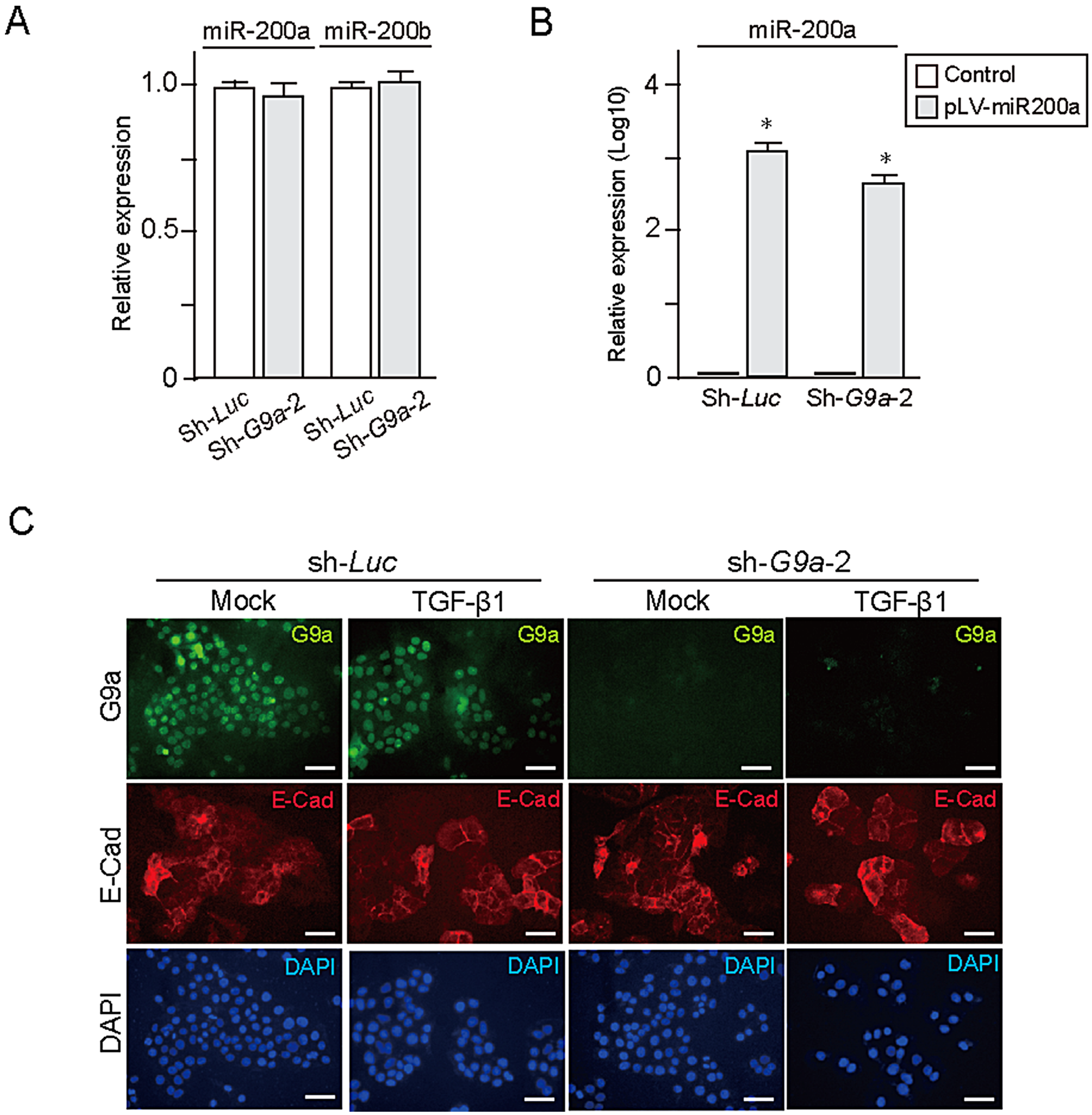

Supplementary Figure 4: Role of miR-200 in *TGF*-β1-induced EMT in Huh7 cells. (**A**) Expression of miR-200a and 200b in stable *G9a* knockdown cells was analyzed using real-time RT-PCR and normalized to U6. Data sets were obtained from three independent experiments. (**B**) Real-time RT-PCR analysis of miR-200a expression in *G9a* knockdown cells stably expressing miR-200a. Data sets were obtained from three independent experiments. (**C**) Immunocytochemical analyses of G9a (green) and E-cadherin (red) expression in *G9a* knockdown cells with miR-200a overexpression. Nuclei stained with DAPI (blue) are also shown. Scale bar = 100 μm.

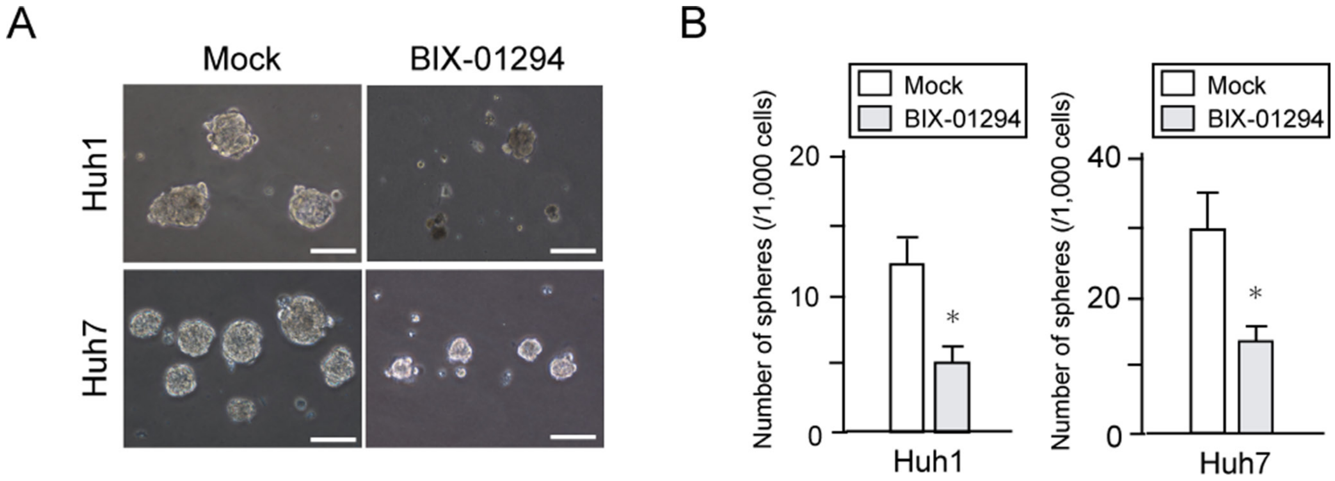

Supplementary Figure 5: Non-adherent sphere formation assay of xenograft tumor-derived cells. (**A**) Bright-field images of tumor cells in non-adherent spheres formed after 14 days of culture. (**B**) Number of large spheres (> 100 μm in diameter) generated from 1,000 tumor-derived cells. Data sets were obtained from three independent experiments. *Statistically significant ($p < 0.05$).

Supplementary Table 1: Potential target genes for G9a

RefSeq	Definition	RefSeq	Definition
C7orf43	chromosome 7 open reading frame 43	HES4	hairly and enhancer of split 4 (drosophila)
FBX02	F-box protein 2	CLIC3	chloride intracellular channel 3
TMEM82	transmembrane protein 82	MTRNR2L9	MT-RNR2-like 9
CADM1	cell adhesion molecule 1	SLC51A	solute carrier family 51 alpha subunit
ISM2	isthmin 2 homolog (zebrafish)	TP53INP1	tumor protein p53 inducible nuclear protein 1
BMP1	bone morphogenetic protein 1	CD55	CD55 molecule
SERINC2	serine incorporator 2	AKR1C2	aldo-keto reductase family 1, member C2
SEC14L2	SEC14-like 2 (s.cerevisiae)	YPEL3	yippee-like 3 (drosophila)
IN080B	IN080 complex subunit B	CYP1B1	cytochrome P450, family 1, subfamily B, member 1
VMA7	von Willebrand factor A domein containing 7	TMEM151A	transmembrane protein 151A
C2orf82	chromosome 2 open reading frame 82	CDKN1A	cyclin-dependent kinase inhibitor 1A (p21, Cip1)
ALDH3B1	aldehyde dehydrogenase 3 family,member B1	ACY1	aminoacylase 1
MOGAT3	monoacylglycerol O-acyltransferase 3	SPOCK2	sparc/osteonectin, cwcv and kazal-like domains proteoglycan 2
PPP1R3B	protein phosphatase 1, regukatory subunit 3B	RTN4RL2	reticulon 4 receptor-like 2
LAMB2	laminin,beta 2 (laminin S)	RRAS	related RAS viral (r-ras) oncogene homolog
PLAU	plasminogen activator, urokinase	C19orf33	chromosome 19 open reading frame 33
SYT12	synaptotagmin XII	SOCS2	suppressor of cytokine signaling 2
TFF1	trefoil factor 1	TRIB1	tribbles homolog 1(drosophila)
TIMP3	TIMP metallopeptidase inhibitor 3	ITPR3	inositol 1,4,5-trisphosphate receptor, type 3
KLF2	Kruppel-like factor 2 (lung)	PPP1R13L	protein phosphatase 1, regulatory subunit 13 like
TMEM176B	transmembrane protein 176B	CYP4F3	cytochrome P450, family 4, subfamily F, member 3
CRYL1	crystallin,lambda 1	PLLP	plasmolipin
ACP5	acid phosphatase 5, tartrate resistant	PMP22	peripheral myelin protein 22
ATP284	ATPase, $Ca2^+$ transporting, plasma membrane 4	NDRG4	NDRG family member 4
JUNB	jun B proto-oncogene	KCNMB4	charybdotoxin receptor subunit beta-4
B4GALNT1	beta-1, 4-N-acetyl-galactosaminyltransferase 1	INHBB	inhibin, beta B
GTPBP2	GTP binding protein 2	TIMP1	TIMP metallopeptidase inhibitor 1
ZFP36	zinc finger protein 36, C3H type, homolog (mouse)	ADM	adrenomedullin
POLD4	polymerase(DNA directed), delta 4	BHLHE40	basic helix-loop-helix family, member e40
DHRS3	dehydrogenase/reductase (SDR family) member 3	OPTN	optineurin
CAB39L	calcium binding protein 39-like	TUBB3	tubulin, beta 3 class III
MAPK11	mitogen-activated protein kinase 11	SPON2	spondin 2, extracellular matrix protein
CXCL16	chemokine (C-X-C motif) ligand 16	NEU4	neuraminidase 4
OLFM2	olfactomedin 2	COL7A1	collagen, type VII, alpha 1
LOXL2	lysyl oxidase-like 2	LAMA3	laminin, alpha 3
LIF	leukimia inhibitory factor	UAP1L1	UDP-N-acetylglucosamine pyrophosphorylase 1 like 1
MTSS1	metastasis suppressor 1	SOCS3	suppressor of cytokine signaling 3
TSKU	tukushi small leucine rich proteoglycan homolog (xenopus laevis)	SAA1	serum amyloid A1
KRT19	keratin 19	DKK3	dickkopf 3 homolog (xenopus laevis)
PNRC1	proline-rich nuclear receptor coactivator 1	SHH	sonic hedgehog
ANGPTL4	angiopoietin-like 4	MXD1	MAX dimerization protein 1
SEZ6L2	seizure related 6 homolog (mouse)-like 2	FGF2	fibroblast growth factor 2 (basic)
TAPBP	TAP binding protein (tapasin)	EGLN3	egl-9 family hypoxia inducible factor 3
KLF4	Kruppel-like factor 4	ADM2	adrenomedullin 2
TRPV2	transient receptor potential cation channel, subfamily V, member 2	LYPD6B	LY6/PLAUR domain containing 6B
PDGFRA	platelet-derived growth factor receptor, alpha polypeptide	PDLIM2	PDZ and LIM domain2
IGFBP1	insulin-like growth factor binding protein 1	C10orf91	chromosome 10 open reading frame 91
MICAL1	microtubule associated monoxygenase, calponin and LIM domain containing 1	AGRN	agrin

Supplementary Table 2: Primer sequences designed for real-time RT-PCR

Gene name	Sequence	
G9a	Fw	5′- TTTCCGCATGAGTGATGATG -3′
	Rv	5′- GGGCAGAACCTAACTCCTCTG -3′
E-Cad	Fw	5′- AAGTTTTCCACCAAAGTCACG -3′
	Rv	5′- TGCTTGGATTCCAGAAACG -3′
N-Cad	Fw	5′- GGTGGAGGAGAAGAAGACCAG -3′
	Rv	5'- GGCATCAGGCTCCACAGT -3'
SNAIL	Fw	5′- GCTGCAGGACTCTAATCCAGA -3′
	Rv	5′- ATCTCCGGAGGTGGGATG-3′
FN1	Fw	5′- GCCACTGGAGTCTTTACCACA -3′
	Rv	5′- CCTCGGTGTTGTAAGGTGGA -3′
VIM	Fw	5′- TACAGGAAGCTGCTGGAAGG -3′
	Rv	5′- ACCAGAGGGAGTGAATCCAG -3′
GAPDH	Fw	5′- CTGACTTCAACAGCGACACC -3′
	Rv	5′- TAGCCAAATTCGTTGTCATACC -3′

Fw, Forward, Rv, Reverse.
